# Rare Renal Malignancies: A Case Series Highlighting Uncommon Presentations and Their Management Strategies

**DOI:** 10.7759/cureus.75119

**Published:** 2024-12-04

**Authors:** Annem Haritha, Sri Balram A, N Rama Murthy

**Affiliations:** 1 College of Medicine, Mamata Academy of Medical Sciences, Bachupally, IND; 2 Department of Urology, Mamata Academy of Medical Sciences, Bachupally, IND; 3 Department of Urology, Mamata Medical College, Khammam, IND

**Keywords:** clear cell renal cell carincoma, ectopic kidney, partial nephrectomy, radical nephrectomy, rcc

## Abstract

This case series elucidates renal cell carcinoma characterized by three distinct presentations, necessitating individualized treatment strategies tailored to each specific circumstance. The three cases presented pertain to clear cell renal cell carcinoma. The first case involves a 44-year-old male patient with renal cell carcinoma in an ectopic kidney, an exceedingly rare occurrence with limited literature. An open transperitoneal radical nephrectomy was undertaken. The second case pertains to a 33-year-old male patient with renal cell carcinoma situated in the lower pole of the left kidney for which partial nephrectomy was planned and performed. The third case concerns a 57-year-old male patient with renal cell carcinoma found in the right lower pole of the kidney, involving invasion into the inferior vena cava (IVC). A radical nephrectomy with tumor thrombectomy was meticulously planned and successfully performed.

## Introduction

Renal cell carcinomas are a diverse group of cancers originating from renal tubular epithelial cells that encompass 85% of all primary renal neoplasms [1]. The subtypes are clear-cell renal cell carcinoma, papillary renal cell carcinoma, and chromophobe renal cell carcinoma [1]. The remaining 15% of tumors of the kidney include transitional cell carcinoma, nephroblastoma or Wilms’ tumor, collecting duct tumors, renal sarcomas, and renal medullary carcinomas [1]. The exact cause of renal cell carcinoma is unknown. Clear cell is the most common and contains a cytoplasm rich in glycogen and lipids. This subtype is likely to be associated with 3p deletion [1]. TRACERx (TRAcking Cancer Evolution through therapy {Rx}) renal program studies showed that loss of 3p was the earliest event in renal cell carcinoma ontogeny, likely occurring in adolescence [2]. Therefore, vigilant monitoring of at-risk patients can facilitate early detection and treatment, leading to improved prognosis. In this case series, we present one case showcasing renal cell carcinoma in an ectopic kidney, a notably rare occurrence alongside two instances of renal cell carcinoma affecting conventional renal anatomy. Ectopic kidneys can be found in the pelvis, iliac region, abdomen, thorax, and crossed and exhibit disease susceptibility similar to that of normally positioned kidneys. However, renal cell carcinoma in an ectopic kidney is exceedingly rare warranting a multidisciplinary approach for diagnostic endeavors compared to that of normally placed kidneys.

## Case presentation

Case 1

**Figure 1:**
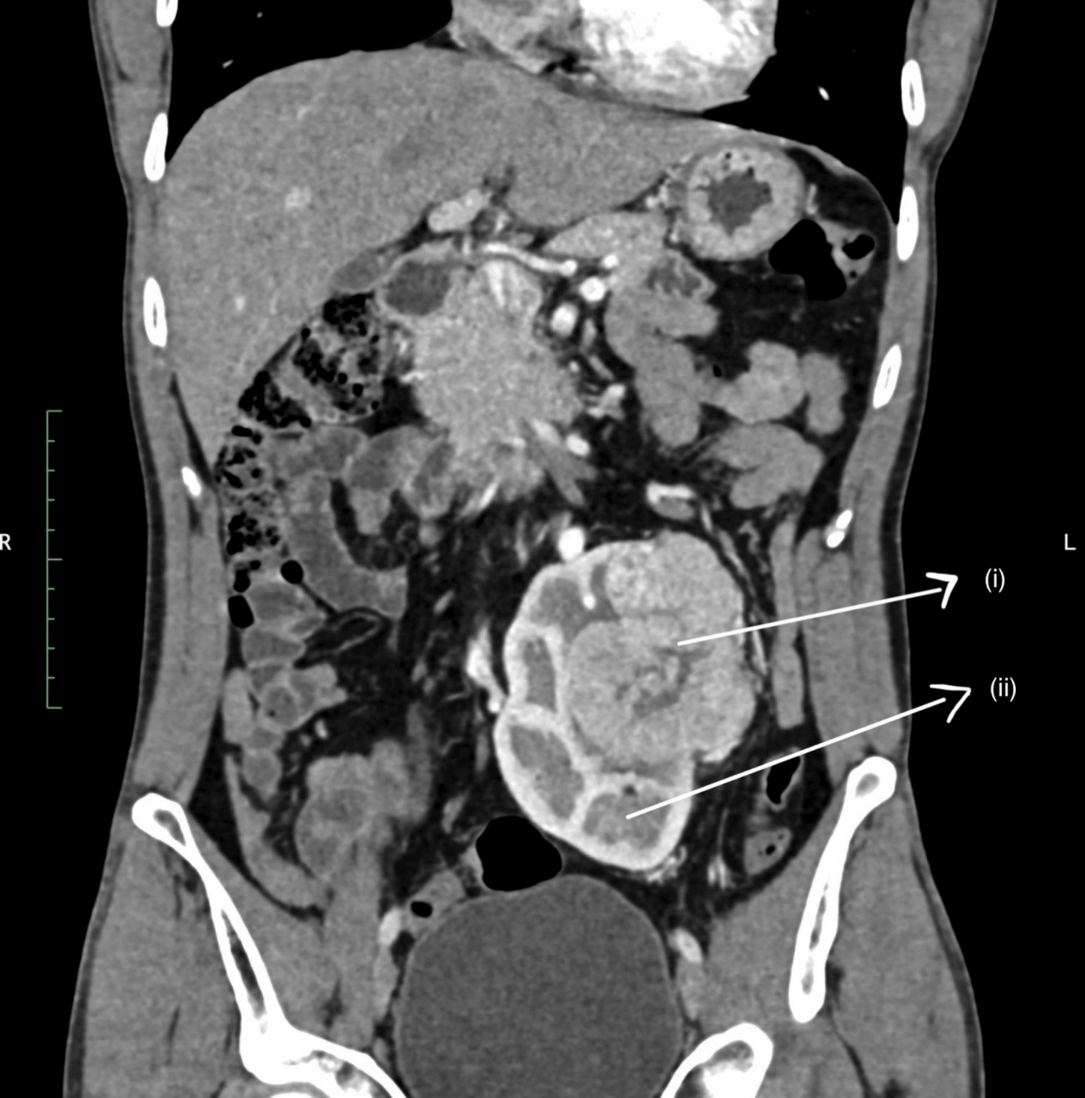
Intense heterogeneous enhancement within the mass; (i) tumor tissue, (ii) normal renal tissue.

A 44-year-old individual with a history of chronic alcoholism presented with persistent, dull aching left flank pain lasting for 10 days. The pain was unaccompanied by fever, dysuria, or other identifiable comorbidities. A palpable abdominal mass was noted upon examination and laboratory assessments revealed microscopic hematuria. Imaging studies unveiled a left iliac ectopic kidney harboring an exophytic mass with marked heterogeneous enhancement (Figure 1).

**Figure 2:**
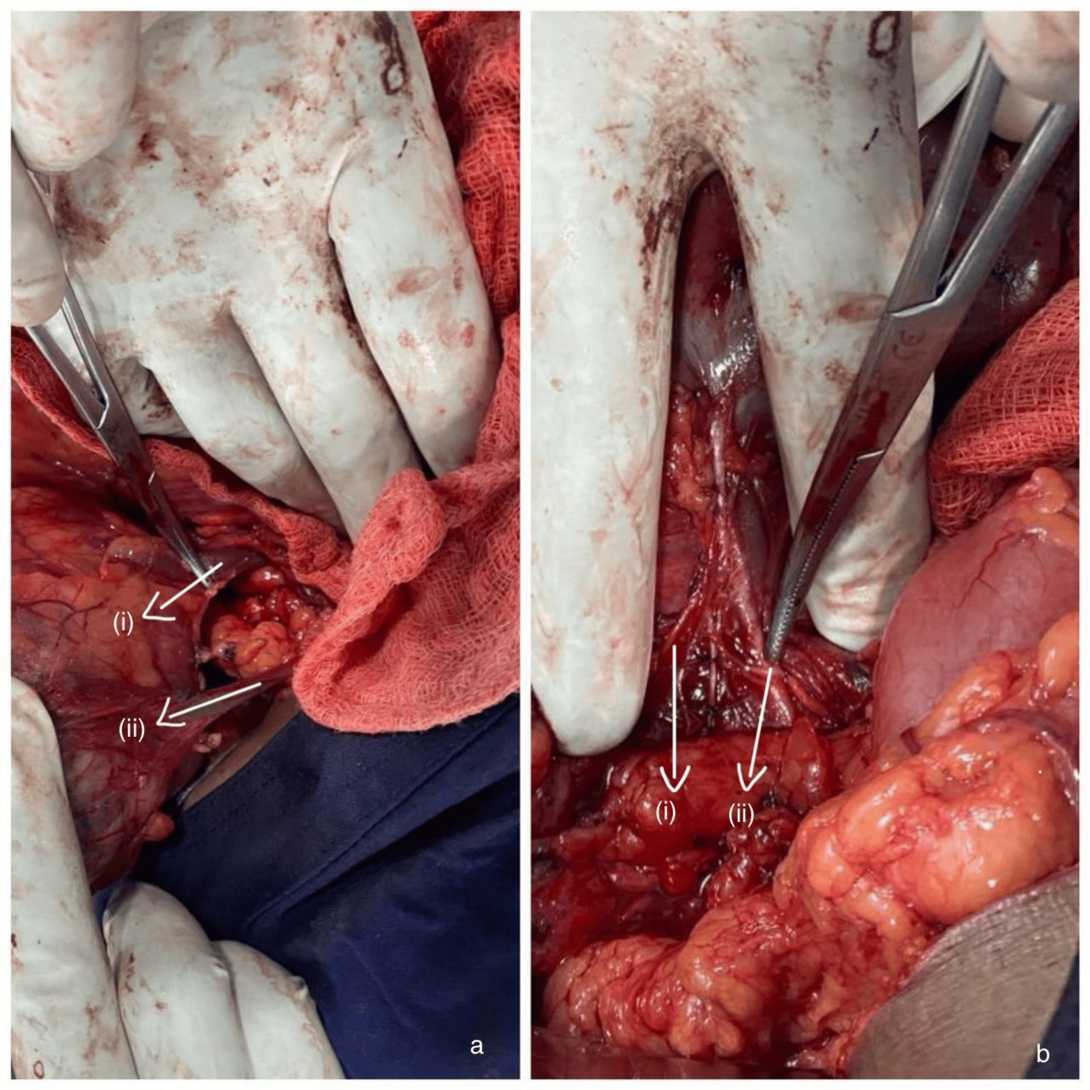
Intraoperative images showing (a) two renal veins, (i) renal vein into inferior vena cava, and (ii) renal vein into left common iliac vein; and (b) two renal arteries arising from (i) left common iliac artery, and (ii) right common iliac artery respectively.

A meticulously planned left-open transperitoneal radical nephrectomy was undertaken under general anesthesia, employing a comprehensive multidisciplinary approach. Notably, the ectopic kidney featured a unique vascular anatomy, with two discernible renal veins (Figure 2a), one terminating into the inferior vena cava and one into the left common iliac vein. Additionally, the presence of two renal arteries (Figure 2b) originating from the right and left common iliac arteries, respectively, further underscored the intricate vascular landscape. Histopathology revealed clear-cell renal cell carcinoma classified as pT2a, Nx, Mx. The patient was under follow-up for two years with no significant complaints or signs of recurrence.

Case 2

**Figure 3:**
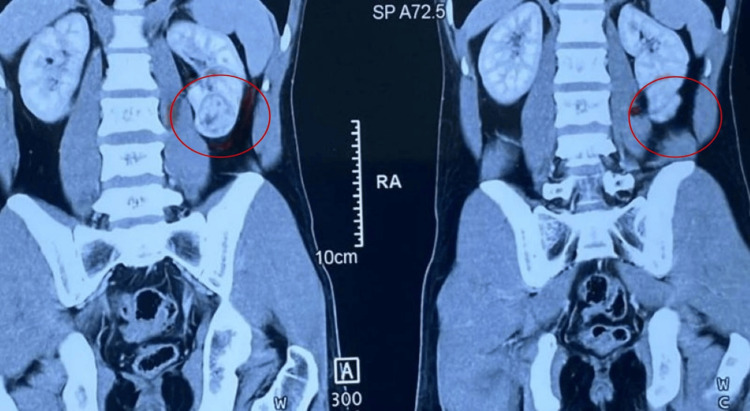
Contrast-enhanced CT showing partially exophytic heterogeneously enhancing lesion.

A 33-year-old male presented with non-specific abdominal pain with no other complaints. Ultrasonography incidentally revealed a well-defined echogenic lesion in the left lower pole of the kidney and subsequent contrast-enhanced computed tomography showed a partially exophytic, heterogeneously enhancing lesion (Figure 3) without involvement of the inferior vena cava or renal veins. The diethylenetriamine pentaacetic acid (DTPA) scan indicated preserved renal function.

**Figure 4:**
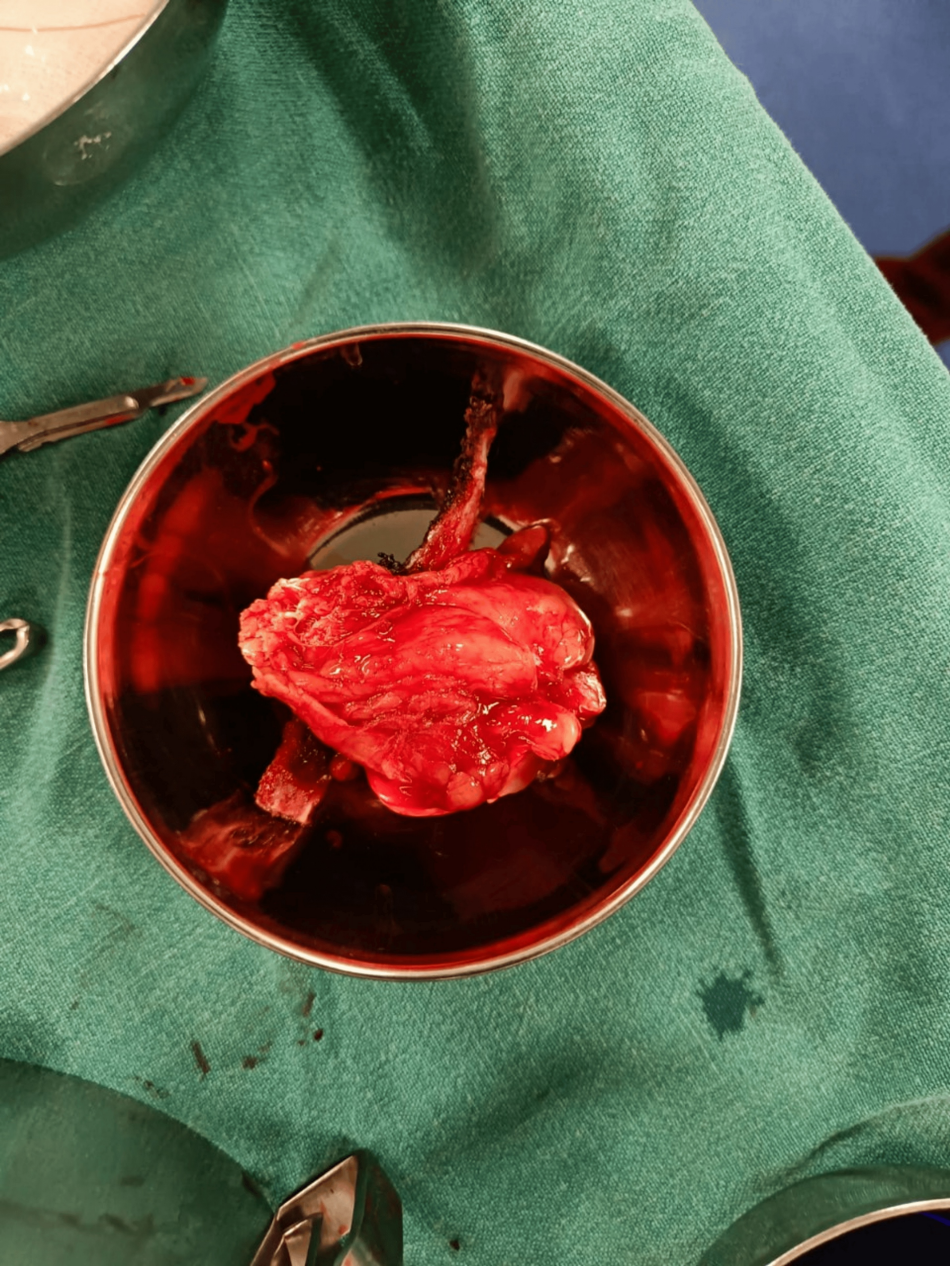
Post-partial nephrectomy specimen.

A meticulous partial nephrectomy (Figure 4) was planned and performed, completely excising the tumor mass located at the lower pole and renorrhaphy was done. Histopathological examination revealed clear cell renal cell carcinoma staged as pT1a, Nx, Mx, confined solely to the kidney with no evidence of lymphovascular invasion.

Case 3

**Figure 5:**
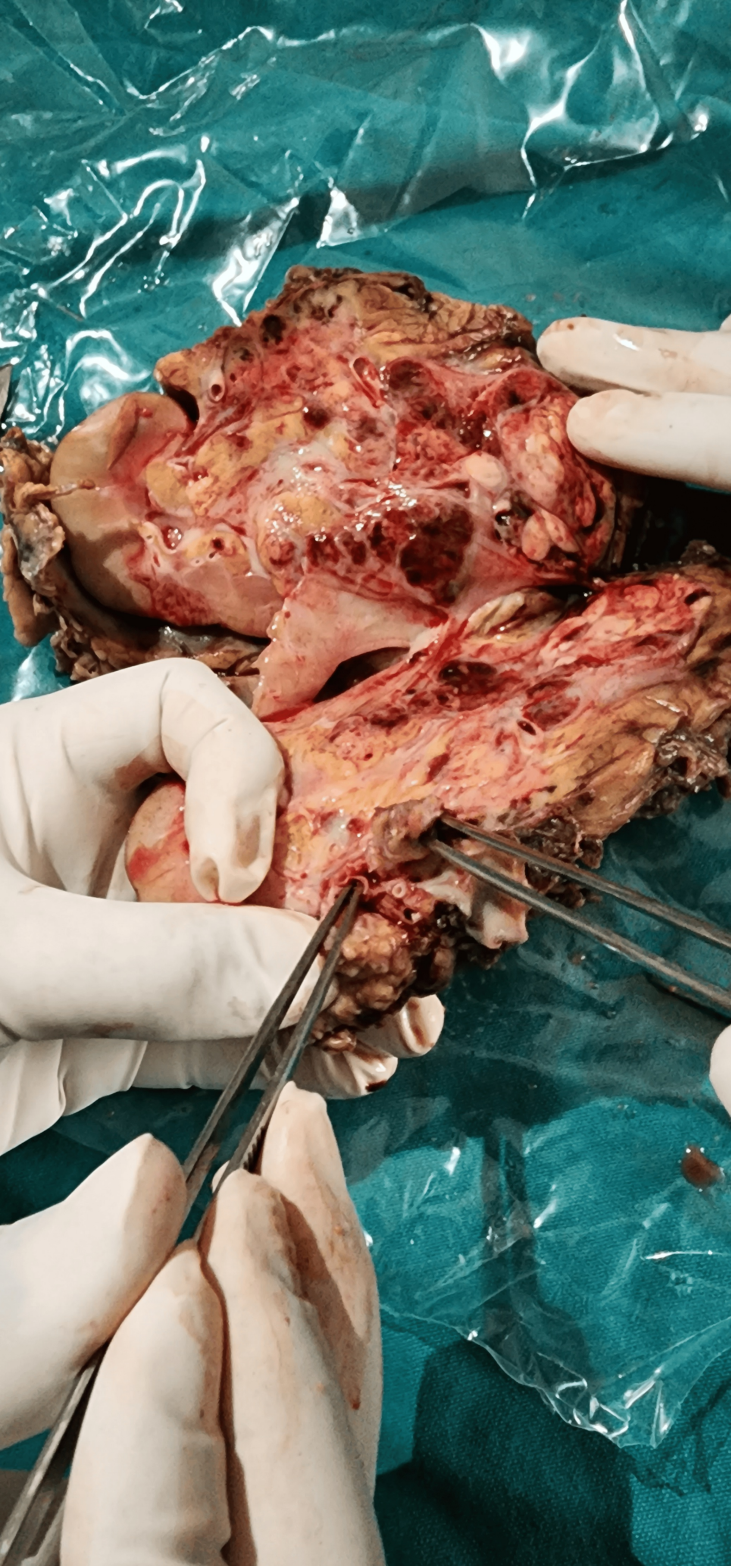
Post-nephrectomy specimen showing the renal vein and section of the inferior vena cava.

A 57-year-old male patient presented with a history of hematuria lasting for two months, without any other complaints. He has a medical history significant for hypertension, and diabetes, and has undergone percutaneous transluminal coronary angioplasty and coronary artery bypass grafting procedures. Imaging studies revealed a large, lobulated, heterogeneously enhanced mass located in the mid and lower poles of the right kidney, with exophytic growth invading the right renal vein and inferior vena cava. Split renal function assessment indicated reduced function of the right kidney. Considering the patient's existing medical conditions and the extent of tumor involvement, radical nephrectomy with tumor thrombectomy was meticulously planned and successfully performed (Figure 5) by placing a temporary inferior vena cava filter to prevent metastasis. Histopathological examination of the specimen confirmed renal cell carcinoma with focal tumor invasion, classified pathologically as pT3a. 

## Discussion

Renal cell carcinoma encompasses a spectrum of cancers arising in the kidney, each possessing distinct histological, biological, and clinical characteristics. The majority of the newly diagnosed renal masses are asymptomatic and no more than 7 cm in diameter [3]. Surgical removal is regarded as the standard treatment for patients with renal cell carcinoma, as the tumor is resistant to chemotherapy and radiotherapy [4].

While the aforementioned cases involve renal cell carcinomas, renal cell carcinoma in an ectopic kidney is exceedingly rare presenting unique challenges in diagnosis and management. There remains ongoing debate among surgeons regarding whether partial or radical nephrectomy represents the optimal choice. The utilization of partial nephrectomy has notably increased in the last decade. Patients undergoing radical nephrectomy usually have more comorbidities and higher risk factors, and experience higher perioperative morbidity and mortality, increased duration of hospital stay, and higher in-hospital costs than patients undergoing partial nephrectomy [3]. Radical nephrectomy is the preferred treatment for tumors where partial nephrectomy is not possible [5].

In the first case, radical nephrectomy was chosen because dynamic renal scintigraphy indicated severely reduced perfusion and cortical uptake in the kidney. This was coupled with cortical thinning and sluggish pelvicalyceal drainage, suggesting minimal remaining kidney function. Completely removing the kidney along with the tumor was deemed the optimal strategy to prevent recurrence, given the compromised state of the kidney. In the second case, due to the patient's young age, absence of other medical conditions, well-preserved renal function, and well-encapsulated tumor mass, partial nephrectomy was favored. The lesion was localized to the lower pole of the kidney, and the remaining kidney function was robust. Given the increasing preference for partial nephrectomy over radical nephrectomy in recent years, partial nephrectomy was chosen as it offers distinct advantages, making it the preferred approach for this patient. In the third case, due to the extensive involvement of the kidney with tumor thrombus extending into the renal vein and inferior vena cava, along with multiple comorbidities and the patient's advanced age, radical nephrectomy was deemed the preferred treatment option. Surgeons can preoperatively estimate the final glomerular filtration rate with radical nephrectomy, based on a scan for split renal function considering 10-20% compensatory hypertrophy post-surgery [6]. Thus, our decision was supported by the split renal function report, which indicated good functioning of the other kidney, thus making radical nephrectomy appropriate and feasible.

It's also noteworthy from this series that not all renal tumors manifest with hematuria: in our cases, the first patient presented with pain and microscopic hematuria, the second had no specific symptoms and the finding was incidental, while the third case presented with visible hematuria. Therefore, clinicians must maintain a high index of suspicion for tumors even in the absence of typical symptoms and select the appropriate treatment tailored to each patient's unique needs.

## Conclusions

Not all cases of renal cell carcinoma exhibit typical symptoms, underscoring the importance of caution in diagnosis and treatment. Caution should be exercised when managing rare cases like renal cell carcinoma in an ectopic kidney due to the diagnostic challenges posed by anatomical anomalies and aberrant vascular supply. Additionally, vigilant monitoring of high-risk patients can aid in early detection and improve prognosis following treatment. The selection between partial nephrectomy (PN) and radical nephrectomy (RN) should be based on individual case characteristics and advanced techniques such as split renal function assessment can be employed as necessary. Further research and studies are needed to determine whether PN offers advantages over RN, particularly in cases where tumors exceed the cT1a classification.
